# Supplementary material for: The Triglyceride-Glucose Index Is Associated with Longitudinal Cognitive Decline in a Middle-Aged to Elderly Population: A Cohort Study
Source: J Clin Med. 2022 Dec 1;11(23):7153. doi: 10.3390/jcm11237153 (PMC9737091; doi:10.3390/jcm11237153)
Supplement: Supplementary file 1 [file jcm-11-07153-s001.zip › jcm-2031780-supplementary.pdf]

Table S1. Baseline characteristics of included vs excluded participants in this study

Characteristic	Total	Included patients	Excluded patients	*p* value
N, (%)				
Age, year, (mean±SD)	54.20±8.92	53.48±8.47	54.89±9.28	<0.001
Sex, n (%)				0.037
Male	1799(49.74)	851(47.97)	948(51.44)	
Female	1818(50.26)	923(50.77)	895(49.23)	
Educational level, n (%)				0.098
Illiterate	144(4.02)	65(3.66)	79(4.37)	
Primary	218(6.08)	95(5.36)	123(6.80)	
Junior or above	3221(89.90)	1614(90.98)	1607(88.83)	
Body mass index, kg/m², (mean±SD)	24.99±84.52	25.02±85.39	24.96±3.40	0.714
Current smoking, n (%)	873(24.14)	394(22.21)	479(25.99)	0.008
Current drinking, n (%)	1138(32.14)	553(31.78)	585(32.48)	0.656
Regular physical activity, n (%)	1009(60.86)	883(53.26)	126(7.60)	0.014
Medical history, n (%)				
Hypertension	1376(38.04)	632(35.63)	744(40.37)	0.003
Diabetes mellitus	514(14.21)	226(12.74)	288(15.63)	0.013
Dyslipidemia	2042(56.46)	1012(57.05)	1030(55.89)	0.482
Laboratory test, (mean±SD)				
TyG index	8.32±0.62	8.31±0.62	8.33±0.62	0.362
FBG, mg/dL	6.28±1.55	6.21±1.42	6.35±1.68	0.006
LDL, mg/dL	3.43±0.80	3.42±0.82	3.44±0.78	0.293
HDL, mg/dL	1.26±0.28	1.27±0.27	1.26±0.28	0.218
TC, mg/dL	5.16±0.96	5.15±0.98	5.17±0.95	0.567
TG, mg/dL	1.99±1.49	2.00±1.53	1.98±1.46	0.599

Abbreviations: Q, quartile; TyG, triglyceride-glucose; FBG, fasting blood glucose; TC, total cholesterol; TG, triglycerides; HDL, high-density lipoprotein; LDL, low-density lipoprotein levels.

Table S2. Subgroups analysis: Risk of cognitive decline based on the increasing the TyG quartiles with various clinical variables

Characteristics	OR (95% CI)				*p* for interaction
	Q1	Q2	Q3	Q4	
Age, years					0.438
< 60	Ref.	1.37(0.93-2.01)	1.41(0.92-2.01)	1.58(1.02-2.45)	
≥60	Ref.	0.90(0.50-1.64)	1.24(0.68-2.26)	1.61(0.84-3.10)	
Sex					0.028
male	Ref.	0.89(0.53-1.49)	1.15(0.70-1.88)	1.39(0.83-2.32)	
female	Ref.	1.51(1.00-2.31)	1.55(0.96-2.51)	1.75(1.05-2.91)	
Body mass index					0.446
< 25	Ref.	1.14(0.76-1.70)	1.24(0.80-1.92)	2.15(1.33-3.47)	
≥25	Ref.	1.21(0.68-2.14)	1.46(0.84-2.53)	1.26(0.72-2.20)	
Hypertension					0.472
Yes	Ref.	0.71(0.37-1.33)	1.42(0.77-2.61)	1.43(0.77-2.66)	
No	Ref.	1.42(0.98-2.07)	1.17(0.77-1.77)	1.50(0.97-2.34)	
Diabetes mellitus					0.137
Yes	Ref.	1.37(0.20-9.59)	3.55(0.56-22.69)	3.37(0.54-20.92)	
No	Ref.	1.19(0.85-1.65)	1.24(0.87-1.76)	1.50(1.02-2.19)	
Dyslipidemia					0.135
Yes	Ref.	1.55(0.74-3.24)	1.40(0.71-2.76)	1.82(0.92-3.60)	
No	Ref.	1.03(0.71-1.49)	2.37(1.24-4.53)	3.99(0.40-40.22)	

The ORs were adjusted for the variables in model 2 in Table 3. ORs for TyG index and the risk of cognitive decline were stratified by age, sex, BMI, hypertension, dyslipidemia and diabetes mellitus.
